# Comparing feature selection and machine learning approaches for predicting *CYP2D6* methylation from genetic variation

**DOI:** 10.3389/fninf.2023.1244336

**Published:** 2024-02-21

**Authors:** Wei Jing Fong, Hong Ming Tan, Rishabh Garg, Ai Ling Teh, Hong Pan, Varsha Gupta, Bernadus Krishna, Zou Hui Chen, Natania Yovela Purwanto, Fabian Yap, Kok Hian Tan, Kok Yen Jerry Chan, Shiao-Yng Chan, Nicole Goh, Nikita Rane, Ethel Siew Ee Tan, Yuheng Jiang, Mei Han, Michael Meaney, Dennis Wang, Jussi Keppo, Geoffrey Chern-Yee Tan

**Affiliations:** ^1^Computational Biology, National University of Singapore, Singapore, Singapore; ^2^Singapore Institute for Clinical Sciences (SICS), Agency for Science, Technology and Research (A*STAR), Singapore, Singapore; ^3^Bioinformatics Institute (BII), Agency for Science, Technology and Research (A*STAR), Singapore, Singapore; ^4^KK Women's and Children's Hospital, Singapore, Singapore; ^5^Duke NUS Medical School, Singapore, Singapore; ^6^National University Hospital, Singapore, Singapore; ^7^Yale-NUS College, Singapore, Singapore; ^8^Institute of Mental Health,Singapore, Singapore; ^9^National Heart and Lung Institute, Imperial College London, London, United Kingdom

**Keywords:** personalised medicine, genomics, epigenetics, *CYP2D6*, machine-learning

## Abstract

**Introduction:**

Pharmacogenetics currently supports clinical decision-making on the basis of a limited number of variants in a few genes and may benefit paediatric prescribing where there is a need for more precise dosing. Integrating genomic information such as methylation into pharmacogenetic models holds the potential to improve their accuracy and consequently prescribing decisions. Cytochrome P450 2D6 (*CYP2D6*) is a highly polymorphic gene conventionally associated with the metabolism of commonly used drugs and endogenous substrates. We thus sought to predict epigenetic loci from single nucleotide polymorphisms (SNPs) related to *CYP2D6* in children from the GUSTO cohort.

**Methods:**

Buffy coat DNA methylation was quantified using the Illumina Infinium Methylation EPIC beadchip. CpG sites associated with *CYP2D6* were used as outcome variables in Linear Regression, Elastic Net and XGBoost models. We compared feature selection of SNPs from GWAS mQTLs, GTEx eQTLs and SNPs within 2 MB of the *CYP2D6* gene and the impact of adding demographic data. The samples were split into training (75%) sets and test (25%) sets for validation. In Elastic Net model and XGBoost models, optimal hyperparameter search was done using 10-fold cross validation. Root Mean Square Error and R-squared values were obtained to investigate each models’ performance. When GWAS was performed to determine SNPs associated with CpG sites, a total of 15 SNPs were identified where several SNPs appeared to influence multiple CpG sites.

**Results:**

Overall, Elastic Net models of genetic features appeared to perform marginally better than heritability estimates and substantially better than Linear Regression and XGBoost models. The addition of nongenetic features appeared to improve performance for some but not all feature sets and probes. The best feature set and Machine Learning (ML) approach differed substantially between CpG sites and a number of top variables were identified for each model.

**Discussion:**

The development of SNP-based prediction models for CYP2D6 CpG methylation in Singaporean children of varying ethnicities in this study has clinical application. With further validation, they may add to the set of tools available to improve precision medicine and pharmacogenetics-based dosing.

## Introduction

Pharmacogenetics based on variants in a few genes has been shown to improve outcomes and cost-effectiveness in drug prescribing (Oates and Lopez, 2018). These established variants are unlikely to fully account for the genetic contributions to individual drug responses. Applying machine learning approaches to genomics data such as epigenetics has the potential to improve the accuracy of predictions and in turn to improve clinical decision-making (Rauschert et al., 2020). This may have value in paediatric drug dosing, where there is less room for error and therapeutic mishaps may have greater consequences (de Beaumais and Jacqz-Aigrain, 2018; Tansuwannarat et al., 2022).

Cytochrome P450 (CYPs) enzymes are a family of proteins involved in metabolism. Cytochrome P450 2D6 (*CYP2D6*) is a subfamily of CYP enzymes that metabolises approximately 25% of commonly used drugs (Shen et al., 2007). *CYP2D6* is highly polymorphic, resulting from different genetic variations such as single-nucleotide polymorphisms (SNPs) and structural variants (Beoris et al., 2016). This variability results in significant interindividual variability in drug reactions and drug efficacy. As such, *CYP2D6* alleles have been found to substantially influence enzyme activity, with *CYP2D6* metabolizer status broadly categorised into poor metabolizers, intermediate metabolizers, extensive (normal) metabolizers and ultra-rapid metabolizers. *CYP2D6* allele frequency is also known to vary among ethnic groups, with Asians and Pacific Islanders having a higher frequency of reduced function allele CYP2D6*10 (Bradford, 2002) leading to slower metabolism.

Epigenetics refers to genomic modifications that can influence gene expression and cellular phenotypes without changing the DNA sequence. DNA methylation, which involves the binding of a methyl group to a cytosine at a CpG dinucleotide, is the most well characterised epigenetic modification in humans and can lead to the inactivation or repression of gene expression (Kacevska et al., 2012). Due to recent advances in high-throughput microarray-based technologies (Dedeurwaerder et al., 2011; Pidsley et al., 2016), genome-wide methylation profiling has become a common approach to complement genome-wide association studies (GWAS).

Growing evidence suggests that genetic variation plays a role in the establishment of DNA methylation marks as well (Villicaña and Bell, 2021). DNA methylation profiles have a genetic basis, as indicated by heritability studies (McRae et al., 2014; Gaunt et al., 2016; van Dongen et al., 2016; Nustad et al., 2018) and associations with nearby SNPs revealed through GWAS (Gamazon et al., 2013). Methylation quantitative trait loci (mQTLs) have been identified in various human populations and cell types (Gibbs et al., 2010; Bell et al., 2011; Fraser et al., 2012), and these have been found to overlap with expression quantitative trait loci (eQTLs; Wagner et al., 2014). In addition to gene variants conferring metaboliser status, there are several SNPs that influence expression of CYP2D6 through mechanisms such as DNA methylation (Lonsdale et al., 2013). Several studies have demonstrated that *CYP2D6* has highly variable methylation, which regulates expression and is influenced by SNP variation (Bonder et al., 2014; Habano et al., 2015; He et al., 2015; Park et al., 2015).

Despite extensive research demonstrating links between genetic variation and epigenetics, there are few models that have integrated genetic variation with individual characteristics to predict methylation and gene expression. Machine learning (ML) technology has been applied in genetics and genomics (Libbrecht and Noble, 2015), particularly for genetic prediction, due to the scaling-up of datasets and computing power. Methods that can work well in high dimensions and identify interactions between loci (Cordell, 2009), without assuming additivity, are appealing. ML algorithms have increased predictive abilities for complex disease risk by handling multi-dimensional data (Ho et al., 2019), which is a challenge for traditional statistical methods. This makes SNP-based ML prediction models attractive for precision medicine. In ML, feature selection is crucial to avoid overfitting, where ML model works better on trained data but not newer ‘test’ data (MacEachern and Forkert, 2021), hindering generalisation, and reduces computation time when working with high-dimensional datasets (Chandrashekar and Sahin, 2014). In ML-based genomic prediction, feature selection improves generalisation and reduces dimensionality by narrowing down the number of SNPs and selecting the ones that have much larger effects than others. The main motivations for feature selection were to produce an inexpensive way to identify a disease phenotype based on measured genotypes of a fewer number of SNPs (Libbrecht and Noble, 2015) and reduce computational complexity for ML. Including millions of SNPs as features could result in overfitting and increase computational time, while excluding too many SNPs could discard important information. Thus, we aim to select informative SNPs based on biological understanding.

Elastic Net have been shown to work well with real-world genetic data (John et al., 2022). It can automatically select significant variables, which efficiently resolves the problem caused by collinearity among the predictor SNP variables that can be problematic in standard regression analyses (Draper and Smith, 1998). The Elastic Net penalty balances between ℓ2 ridge-regression penalty and ℓ1 lasso penalty, and the choice of the regularisation parameter (λ) is critical to selecting important variables with accurate estimation. Tuning parameters α and λ are usually chosen based on cross-validations to minimise mean-squared prediction error. Elastic Net algorithms are a modified form of linear regression that reduce overfitting for linear relationships.

XGBoost algorithm, which has been shown to outperform traditional ML algorithms such as linear regression and K Nearest Neighbour in predicting gene expression values (Li et al., 2019) and improving breast cancer risk prediction accuracy based solely on genetic variants (Behravan et al., 2020).

Gradient boosting fits new models consecutively to provide an accurate estimate of the response variable (Natekin and Knoll, 2013) and uses a gradient descent algorithm to minimise the loss when adding new models. At each particular iteration, a new weak, base-learner model is trained with respect to the error of the whole ensemble learnt. XGBoost is a version of gradient boosted trees that has been optimised to perform well in distributed computing environments (Ho et al., 2019), making it highly efficient.

As such, in this study, we focus on examining the best set of SNP features suitable for use in the prediction of *CYP2D6*-associated CpG methylation levels using *CYP2D6* SNP genotypes after various feature selection methods. We also compare the performance of two ML algorithms, Elastic Net (Zou and Hastie, 2005) and eXtreme Gradient Boosting (XGBoost; Chen and Guestrin, 2016) and investigate their performance and prediction accuracy with regard to the different SNP feature sets to identify the optimal method for SNP feature selection.

## Materials and methods

### Growing up in Singapore towards healthy outcomes (GUSTO) cohort

Samples used in this project were taken from GUSTO (Soh et al., 2014), a mother-offspring prospective cohort study in Singapore.

Between 2009 and 2010, the GUSTO study recruited pregnant women who attended their first trimester antenatal dating ultrasound scan clinic at National University Hospital (NUH) and KK Women’s and Children’s Hospital (KKH). A total of 1,247 mothers were recruited such that 55.9% were Chinese, 26.1% Malay and 18.0% Indian, with homogeneous parental ethnic background. A total of 1,176 babies were born in the cohort. Demographic variable information such as ethnicity, mother’s age, education and income were collected at Pregnancy week 11 timepoint. The current study included 414 pregnant mothers. The ethnic distribution of the children, based on the parents, was 57.2% Chinese, 26.1% Malays and 13.5% Indians. Out of the 414 children reported in the study, 194 were female (46.9%) while 210 were males (50.7%). The average age of mothers at recruitment was 30.9 years (*SD =* 5.22). The distribution of participants’ highest education levels varied, with 23 participants having completed primary education (5.6%), 101 participants having completed secondary education (24.4%), 104 participants having attained General Certificate of Education (25.1%), 38 participants having attained a National Technical Certificate (9.2%) and 133 participants having completed tertiary education (32.1%; Table 1). Additional demographic information can be found in Supplementary 1.

**Table 1:** Number of SNPs obtained for each of the feature sets, before and after filtering for MAF > 10%.

Feature set				No. of SNPs obtained before excluding MAF < 10%	Final no. of SNPs obtained after excluding MAF < 10%
1	GWAS – mQTLs from 75% of GUSTO samples	1.1. Before B-H correction	1.1.1. cg04692870-Probe 1	173	129
			1.1.2. cg07016288-Probe 2	234	164
			1.1.3. cg09322432-Probe 3	251	190
			1.1.4. cg10840135-Probe 4	220	149
			1.1.5. cg15597984-Probe 5	219	170
			1.1.6. cg17498424-Probe 6	227	164
			1.1.7. cg20046859-Probe 7	206	153
			1.1.8. cg22650942-Probe 8	199	119
			1.1.9. Combining SNPs for above eight CpG probes (duplicates removed)	1,364	983
		1.2. After B-H correction	1.2.1. cg04692870-Probe 1	2	2
			1.2.2. cg07016288-Probe 2	1	0
			1.2.3. cg09322432-Probe 3	13	11
			1.2.4. cg10840135-Probe 4	2	2
			1.2.5. cg15597984-Probe 5	7	7
			1.2.6. cg17498424-Probe 6	0	0
			1.2.7. cg20046859-Probe 7	1	1
			1.2.8. cg22650942-Probe 8	0	0
			1.2.9. Combining SNPs for above eight CpG probes (duplicates removed)	18	15
2	GTEx database			710	548
3	41.5 Mb – 43.6 Mb range			3,149	2,406

### Child genotype data

Infant DNA obtained from umbilical cord tissue at the Delivery time point as part of the study cohort visit, was genotyped using Illumina OmniExpress plus Exome array, which was performed by Expression Analysis, Inc. This particular array offers more comprehensive representation of genetic variations within Asian populations compared to other commonly used arrays (Jiang et al., 2013). Data were processed using GenomeStudio Genotyping Module version 1.0 developed by Illumina, Inc. The GenCall software makes genotyping calls such that genotypes with a GenCall score lower than 0.15, are not assigned genotypes. Samples that did not meet the criteria of a genotyping call rate of at least 97%, self-reported ethnicity, sex, or had an inconsistent offspring-parent relationship were excluded.

Each ethnic group was subject to genotype imputation separately (scripts are available at the link in Supplementary 2). Briefly, SNPs with minor allele frequency (MAF) < 5%, call rate < 95% or failed Hardy–Weinberg Equilibrium at value of *p* < 10^−6^ were excluded from each ethnicity, using PLINK version 1.90 (Chang et al., 2015). The data was then aligned to the GRCh37 build and processed further using a published pipeline^2^. After further processing, the data was subjected to haplotype phasing using the SHAPEIT2 software with duoHMM method (O’Connell et al., 2014), which takes family structure into account for increased accuracy. The phased haplotypes were then imputed using the PBWT algorithm (Durbin, 2014) and the Sanger Imputation Service (McCarthy et al., 2016), with the 1,000 Genomes Phase 3 (1,000 Genomes Project Consortium 2015) used as the reference panel. A total of 4,869,008 SNPs that passed stringent quality control (MAF > 5% and imputation INFO>0.80) in at least one ethnic group were analysed.

### Child methylation data

DNA methylation profiling on 414 child buffy coat samples obtained at the study visit (Year 6 time point) when the infants turned 6 years old, was performed using Infinium MethylationEPIC BeadChip (850 K; ‘EPIC 850 K’). DNA methylation IDAT files were read using the *minfi* R package (Aryee et al., 2014). Probes that did not meet standard protocol and quality control procedures were removed. The removal criteria included having less than three beads for any sample or a signal detection value of p (based on the signal compared to background for each bead intensity) greater than 0.01 for any sample. Probes from sex chromosomes were also removed. Within-sample normalisation was performed using Noob pre-processing (Triche et al., 2013).

For each CpG site, the percentage of methylation was computed by dividing the intensity of the methylated probe by the overall intensity of the CpG site. This produced a value between 0 and 1. Methylation beta values were first converted to M-values before applying COMBAT to remove batch effects (Johnson et al., 2007). Subsequently, the batch-corrected methylation values were transformed back to beta values. Finally, probes that exhibited cross-hybridisation (Chen et al., 2013; Price et al., 2013) and probes with a methylation range (maximum-minimum, excluding outliers) of less than 5%, were excluded from the analysis. In total, 440,567 CpGs passed the QC criteria. Genome coordinates (hg19 build) and gene annotations of these CpGs were extracted from the Infinium MethylationEPIC BeadChip manifest file V1.0 B4 (Illumina, 2017).

### Selection of *CYP2D6* CpG sites

Genetic prediction models were trained to predict child *CYP2D6* CpG status (methylation beta value). The detailed annotation table, including the chromosomal position can be found in Supplementary Table 3. Eight CpG sites were identified to be annotated under *CYP2D6*, which we have labelled as Probe 1–8 for clarity: cg04692870-Probe 1, cg07016288-Probe 2, cg09322432-Probe 3, cg10840135-Probe 4, cg15597984-Probe 5, cg17498424-Probe 6, cg20046859-Probe 7, cg22650942-Probe 8.

### Feature selection – SNPs

To identify informative SNP features for predicting *CYP2D6* methylation status, we employed three methods. First, we used GWAS to identify SNPs associated with *CYP2D6* methylation status as traits. Next, we used *CYP2D6* eQTLs from GTEx which provided independent and relevant information on methylation. Lastly, we used a range of SNPs within 2 Mb of the *CYP2D6* gene (Dimas et al., 2009) to examine if including more SNPs with varying information could improve ML performance.

The respective genetic information for each set of SNPs was filtered from GUSTO genotype data using PLINK 1.9 – *recode* and – *extract* function. SNPs with less than 10% minor allele frequency (MAF) were removed as per various GWAS studies methods (Florez et al., 2007; Tabangin et al., 2009) for quality control. 10% MAF was chosen due to power limitations in genetic association studies, as rare variants require large sample sizes to be studied.

#### Feature set 1 – mQTLs from 75% of GUSTO samples

To test our hypothesis that highly statistically associated SNPs with DNA methylation (mQTLs) as features improves prediction models, we conducted GWAS on the eight *CYP2D6*-associated CpG sites. mQTLs could act as important cis-regulatory polymorphisms connecting genetic variation to methylation variation and have been linked to regulatory functions and disorders (Lin et al., 2020). mQTL identification involves association tests between genome-wide genetic variation and DNA methylation levels at specific CpG sites (Villicaña and Bell, 2021). Methylation can serve as the phenotype of interest for GWAS.

We obtained mQTLs from 75% of the available samples of each CpG site. Conducting GWAS on all samples with methylation data could lead to biased model performance due to data leakage. Applying GWAS to 75% of the samples ensures mQTLs of the 25% test validation set were not selected as features.

#### Feature set 1.1 – derived from genome-wide association study to identify *CYP2D6* mQTLs

GWAS analysis was conducted separately for each of the eight CpG sites. For example, to obtain mQTLs for cg04692870-Probe 1, GWAS was performed on 75% of the available samples (300 out of 401) using PLINK 1.9. We performed LD pruning using the variant pruning tool (*-indep* 500 5 2) in PLINK 1.9, resulting in 247,574 remaining SNPs. Principal component analysis (PCA) was also conducted in PLINK 1.9, and the top five principal components (PCs) were chosen as covariates for GWAS based on the scree plot of eigenvalues. Basic linear regression style GWAS was then run on the child genotype data of the 300 samples using the *--linear* function in PLINK 1.9, and the value of p was calculated for any significant SNP rsIDs correlated with cg04692870-Probe 1.

We applied two statistical thresholds to obtain feature sets for analysis. A larger set of SNPs (Feature set 1.1 – Before B-H correction) were selected with a filter of SNPs from chromosome 22 with an uncorrected value of *p*<0.05. We decided to include features selected with uncorrected thresholds in our analysis as other studies have demonstrated superior performance at lower thresholds in polygenic risk scores (PRS) as well as machine learning models (Fergus et al., 2018; Privé et al., 2019). This was repeated for each of the eight CpG sites (Feature sets 1.1.1 to 1.1.8 for CpG Probes 1–8 respectively). Additionally, all SNPs found significant to their respective CpG sites were combined across all CpG sites, with duplicated SNPs removed (Combined Feature set 1.1.9).

#### Feature set 1.2 – after B-H correction

Further GWAS analysis includes correction for multiple testing. The significance threshold used for SNP–trait associations was the Benjamini–Hochberg (B-H) false discovery rate (FDR) of value of *p* < 0.05 which was chosen to minimise the expected proportion of false positives (Benjamini and Hochberg, 1995). Only significant SNPs on chromosome 22 were considered. This was repeated for each of the eight CpG sites (Feature sets 1.2.1 to 1.2.8 for CpG Probes 1–8 respectively). All SNPs found significant to their respective CpG sites after B-H correction were also combined across all relevant CpG sites, with duplicated SNPs removed (Combined Feature set 1.2.9).

#### Feature set 2 – genotype-tissue expression database

The Genotype-Tissue Expression (GTEx) database was utilised to obtain *CYP2D6*-associated SNPs independent of methylation data. The GTEx database is an established resource to study the relationship between genetic variants and gene expression in multiple human tissues, with samples collected from 54 non-diseased tissue sites across nearly 1,000 individuals. Single-tissue eQTLs for *CYP2D6* in all tissues were obtained from the GTEx database, filtered for duplicates and those present in the GUSTO child genotype data using PLINK 1.9. This allowed access to additional samples that support independent discoveries, and for unbiased eQTLs to be used as features for ML.

#### Feature set 3–41.5 to 43.6 Mb range from GTEx database

To capture potential SNPs that may affect *CYP2D6* expression and methylation, a wider range on chromosome 22 was explored, given that some SNPs could be located far upstream or downstream of the gene (Schadt et al., 2008; Yang et al., 2010; Wang et al., 2014). Significant single-tissue eQTLs for *CYP2D6* from the GTEx database were used as a reference to obtain the smallest (rs116099340 at position 41,133,140) and largest (rs151076151 at position 43,107,039) position of the first reference sequence base. The genomic coordinates of these SNPs were obtained from the UCSC Genome Browser (GRCh37/hg19) and used to obtain a list of SNPs in this range (41.5 Mb to 43.6 Mb) from the GUSTO genotype data using PLINK 1.9.

Refer to Table 1 for the list of the Feature sets.

### Statistical analysis

#### Splitting the train and test data

In an initial dataset, all samples were randomly split into two datasets, comprising 75% for training and 25% for the creation of a standard test set (101 samples). This 25% standard test set served as a benchmark for subsequent dataset divisions. For each CpG probe ID (Probes 1–8), samples with the same sample ID as those present within the 25% standard test set will be exclusively assigned to the test set, and the rest of the samples formed the train dataset. This was then used for all analysis.

#### Heritability estimates

We calculated a heritability estimate for each CpGs with significant SNPs after B-H correction, using a pseudo-PRS. PRS was calculated according to the following equation: PRS(CpG) = sum(weight_i * SNP_i). Weight_i is the beta value from GWAS study, SNP_i is the SNP dosage (0,1,2 addictively encoded on minor allele; Zhu and Zhou, 2020). We then calculated the R^2^ on the association between [PRS(CpG) ~ CpG]. The R^2^ is the coefficient determination of genetic factors that contribute to the explanation of CpG changes, and this was calculated for the train set and test set.

#### ANCOVA and linear regression

We analysed for influences of demographic characteristics such as socioeconomic status on CpG methylation data, and this was to determine important demographic variables that may be contributing to methylation in the eight CpG sites. ANCOVAs were conducted using JASP version 0.16.4.0 software (JASP Team, 2022) to investigate statistically significant differences in methylation levels at *CYP2D6* CpG probe sites based on different demographic variables. The demographic variables include mothers’ income, household income, type of accommodation, child’s ethnicity, mothers’ highest education, and child’s sex, while mother’s age during recruitment was entered as a covariate. Using each *CYP2D6* CpG probe site as the dependent variable, the F statistic and value of p for each demographic variable were reported by JASP.

Linear regression models were also used to examine the association of the SNPs’ genotype with each of the eight CpG loci status using JASP. Information on covariates was available for children and their mothers across all eight CpG sites; where relevant, these variables were adjusted for in the statistical models. These variables included the top five PCs of the genotype data, mothers’ age during recruitment, mothers’ income, household income, type of accommodation, child’s ethnicity, mothers’ highest education, and child’s sex.

In the 75% train set, coefficients of each of the SNPs’ genotypes and all covariates in a linear regression were obtained from JASP. The coefficients were then entered into a linear equation in R to predict the *CYP2D6* CpG methylation values in the 25% test set. Root Mean Square Error (RMSE) and R-Squared (R^2^) values were then calculated based on the predicted and actual methylation values in the 25% test set. Regression performance metrics for the training set were reported by JASP. This standard test set was used similarly for the other CpG probes.

This statistical analysis was done only for Combined Feature set 1.2.9 – GWAS of 75% samples after B-H Correction, with all significant SNPs combined (Table 2) due to the small subset of SNPs present in this feature set. It was not feasible to conduct regression analysis in JASP with the larger number of SNPs from the other sets of features.

**Table 2:** Demographics table.

	Descriptive statistics	Effect of demographics on methylation		
	N (%)	Statistic	cg09322432-Probe 3	cg15597984-Probe 5
Child’s Ethnicity		*ANCOVA, F (value of p)*	3.791 (0.024)	5.48 (0.005)
Chinese	237 (57.2)	Mean methylation (SD)	0.902 (0.021)	0.743 (0.031)
Malay	108 (26.1)		0.895 (0.027)	0.737 (0.037)
Indian	56 (13.5)		0.895 (0.018)	0.726 (0.028)
Missing	13 (3.1)			
	Mean (SD)	Significant CpG site	cg17498424-Probe 6	
Age in years at recruitment	30.9 (5.22)	Linear Regression*, B (value of p)*	−6.210 × 10^−4^(0.010)	
	N (%)	Significant CpG site	cg07016288-Probe 2	
Monthly income of mothers SGD		*ANCOVA, F (value of p)*	2.981 (0.019)	
<$1,000	131 (31.6)	Mean methylation (SD)	0.848 (0.032)	
$1,000–1,999	98 (23.7)		0.845 (0.032)	
$2,000–3,999	100 (24.2)		0.843 (0.033)	
$4,000–5,999	26 (6.28)		0.856 (0.031)	
≥$6,000	7 (1.69)		0.817 (0.030)	
Not answered	52 (12.6)			

#### Collinearity between SNPs

LD analysis was performed using Haploview v. 4.1 program (Barrett et al., 2005). The squared correlation (R^2^) between allelic values at two loci were computed. A higher R^2^ shows higher collinearity between two SNPs. This was done for SNPs obtained from Combined Feature set 1.2.9 – GWAS of 75% samples after B-H Correction, with all significant SNPs combined, to ascertain the use of linear models in statistical analysis for small feature sets.

#### Machine learning models and optimisation

To develop ML models of CpG status using cis-acting genetic variation, *CYP2D6* eQTLs and mQTLs feature sets were used as features in Elastic Net and XGBoost models to predict the status of each of the eight CpG sites. Mode imputation was done to replace missing genetic data in samples.

To prevent encountering errors when executing ML algorithms, we conducted preprocessing on both the training and testing datasets. Train and test dataset splitting was done with a standard test set as per aforementioned. To ensure all genotype data of each SNP feature were in the train set, the number of unique genotype categories within each SNP column were compared and columns where the test set exhibited a greater number of unique categories than train set were identified. A randomly selected row with the unique genotype category in the test set was then transferred to the train set. Next, to ensure that the train set consistently contains a minimum of two samples for every category within each SNP column, for columns featuring a category with only one sample, we appended that specific category to a new row within the column. For the remaining columns, we introduced a randomly selected genotype from the existing column data. Lastly, we identified columns containing only two unique values in the test set. For such columns, we introduced a new row, added the missing class found in the train set and filled the remaining columns with random values from their respective distributions. This ensured that all columns in the test set consistently contained at least three unique values, similar to the train set, for the conversion of these datasets to sparse matrices downstream.

Training data was used to obtain an unbiased estimate of the hyperparameters for the best performance of the models via cross-validation, and the test set is used to obtain an unbiased final model performance metric for validation. The average RMSE and R^2^ for the train and test set were reported to find the models’ performances in prediction using the specific sets of features.

#### Elastic net

In our Elastic Net model, we used *glmnet* from R package and *caret* to fit a grid of models to select optimal α and λ parameters jointly through 10-fold cross-validation on the training data. This was repeated five times, using a different set of folds for each cross-validation. The optimal parameters were chosen based on minimising the mean squared error between the predicted and actual values. The resulting model was trained on the training set, and its performance was validated on the test set across the eight CpG probes using RMSE and R^2^. The average RMSE and R^2^, across all predicted CpG sites were reported for each set of features.

#### XGBoost

Elastic Net algorithms are a modified form of linear regression that reduce overfitting if CpG methylation data is linear. To account for non-linear effects, we used the XGBoost algorithm, Our XGBoost model employed one-hot encoding to convert categorical genetic data into a sparse matrix before fitting it into the model using the R package *xgboost*. Hyperparameters were tuned through 10-fold cross-validation with 100 rounds of boosting and early stopping after 100 rounds without improvement in the cross-validation loss. The optimal hyperparameters (max_depth, eta, subsample and colsample_bytree) and best index were obtained based on the minimum test RMSE, which were used to train the model on the training set and predict the CpG loci status on the test set. The performance of the model was evaluated using RMSE and R^2^, and the average values for each set of features were reported.

#### Integration of non-genetic and principal component factors in machine learning models

In addition to using only genetic variants as features, we included non-genetic demographic features to model interactions of genetic variation with demographic factors at CpG loci in children. These features comprised demographics of children with both genotype and methylation data, such as mothers’ age during recruitment, income, household type, ethnicity, education, child’s sex, and the top five principal components (PCs) of the genotype data. The non-genetic factors had varying amounts of missing data, and mode imputation was used to replace missing genetic and categorical non-genetic data, while mean imputation dealt with missing mothers’ age data. Elastic Net and XGBoost models were run as previously mentioned. For each set of genetic features, with non-genetic and PC features included, the average RMSE and R^2^ across all predicted CpGs were reported.

#### Feature importance

Since multiple variables, including non-genetic factors, were employed in predicting CpG methylation, it would be valuable to demonstrate the significance of the top 10 predictors in the best performing model in each CpG probe. This was done by calculating SHapley Additive exPlanations (SHAP) score of each feature in their respective models. Feature importance was ranked, and top 10 features of the best performing models of each CpG probe were obtained.

R codes used can be found at the repository link provided in Supplementary 2.

## Results

### Feature selection

We applied feature selection according to the three methods as proposed in order to obtain different sets of SNP features that can be compared for their performance as features in ML. In total, we obtained 17 sets of features, each consisting of a different number of SNPs deemed to provide important information in ML.

After MAF filtering, we identified 983 mQTLs (Combined Feature set 1.1.9) from a GWAS of the training set comprising 75% of samples at an uncorrected threshold of *p* < 0.05 and 15 mQTLs (Combined Feature set 1.2.9) at a corrected threshold. We also utilised 548 eQTLs (Feature set 2) influencing expression of *CYP2D6* across the body from GTEx and 2,406 (Feature set 3) from a 2 MB range around the gene. As there were no significant SNPs for cg07016288-Probe 2, cg17498424-Probe 6, and cg22650942-Probe 8, they were excluded from analysis to allow comparison in performance between after B-H feature sets (Feature Sets 1.2) and other feature sets.

There were no significant associations with cg07016288-Probe 2, cg17498424-Probe 6, and cg22650942-Probe 8. In general, fewer SNPs remained after excluding SNPs with MAF < 10%.

A complete list of SNP rsIDs obtained from all feature sets methods can be found in Supplementary 4.

### Collinearity between mQTLs

**Figure 1:**
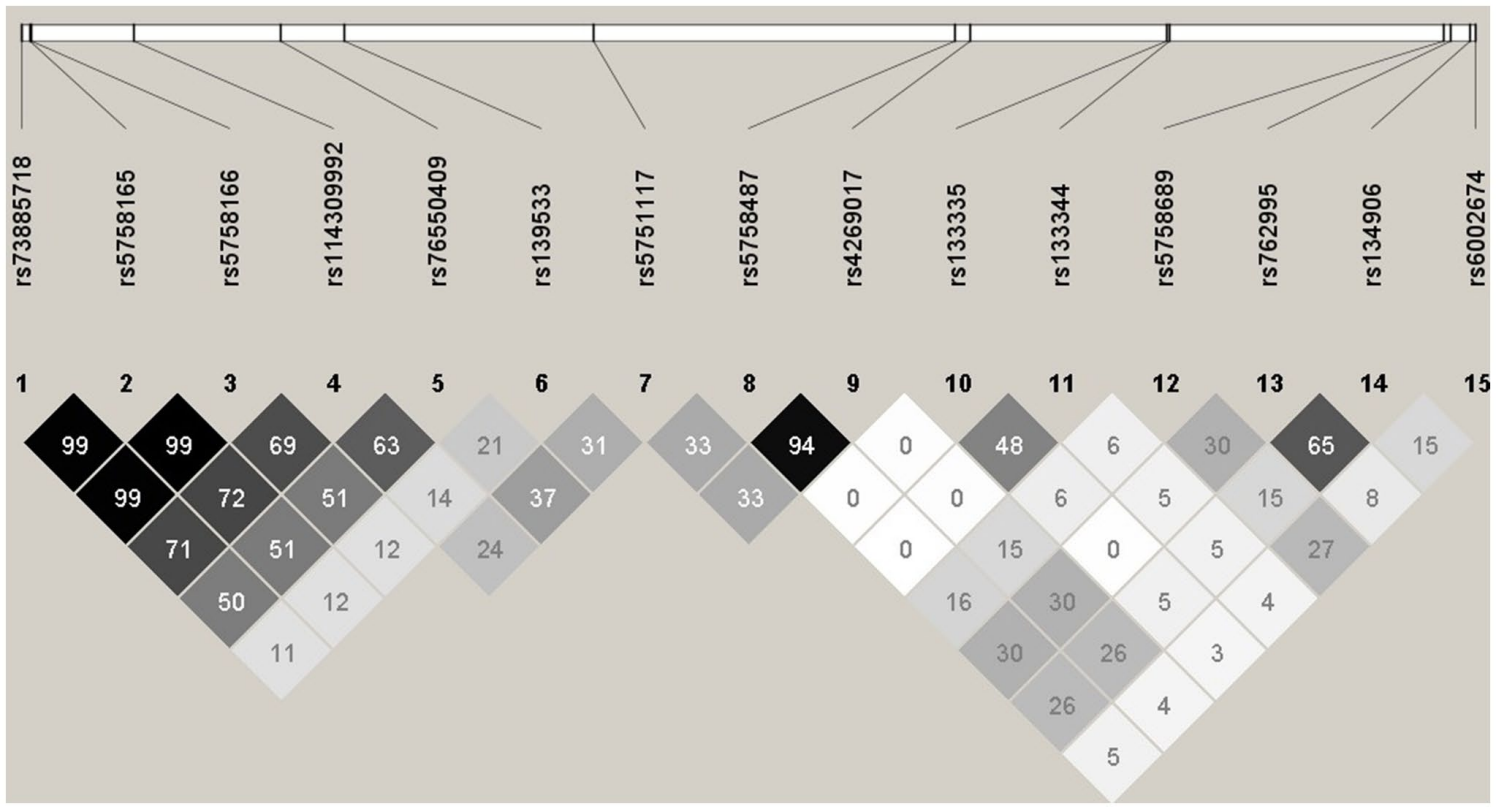
Haploview plot illustrating the linkage disequilibrium of 15 SNPs from Feature set 1.2.9. Numbers indicate the R^2^ values, and grey squares with different intensity indicate 0 < *R^2^* < 1.

LD plot was done for Combined Feature set 1.2.9 – GWAS of 75% samples after B-H Correction, with all significant SNPs combined (15 SNPs). LD analysis revealed high R^2^ at some SNP combinations (Figure 1), indicating high collinearity among some of the SNPs in Combined Feature set 1.2.9. For example, rs73885718 and rs5758165 have high collinearity (*R^2^* = 0.998), and both of these mQTLs are significant in cg09322432-Probe 3.

### Demographic effects

There was a significant effect of child’s ethnicity on 2 CpG sites, cg09322432-Probe 3 [*F*(2, 320) = 3.791, *p* = 0.024] and cg15597984-Probe 5 [*F*(2, 323) = 5.48, *p* = 0.005], after controlling for mother’s age at recruitment. In cg09322432-Probe 3, this appeared to be driven by greater methylation in Chinese as compared to Malays (95% CI [0.002, 0.012], *t*(373) *=* 2.658, *p* = 0.008) and Indians (95% CI [6.355 × 10^−4^, 0.014], *t*(373) *=* 2.155, *p* = 0.032). In cg15597984-Probe 5, Chinese had greater methylation than Indians (95% CI [0.008, 0.027], *t*(373) = 3.540, *p* = < 0.001), and Malays also had greater methylation than Indians (95% CI [7.822 × 10^−4^, 0.027], *t*(373) = 2.110, *p* = 0.036). Additionally, children with younger mothers had greater methylation on cg17498424-Probe 6 [*B* = −0.000621, *t*(320) *=* −2.590, *p* = 0.01]. There was also a significant effect of mother’s monthly income on cg07016288-Probe 2 [*F*(4, 323) = 2.981, *p* = 0.019]. This effect appears to be driven by greater methylation in mothers in lower income groups, specifically with regard to mothers earning more than 6,000 SGD as the reference category. Mothers earning less than 1,000 SGD exhibited greater methylation than mothers earning more than 6,000 SGD (95% CI [0.006, 0.055], *t*(357) = 2.470, *p* = 0.014). Similarly, mothers earning between 1,000 to 1999 SGD had greater methylation than mothers earning more than 6,000 SGD (95% CI [0.004, 0.053], *t*(357) = 2.260, *p* = 0.024). Mothers who earned between 2000 to 3,999 SGD also had greater methylation than mothers earning more than 6,000 SGD (95% CI [0.002, 0.051], *t*(357) = 2.108, *p* = 0.036). Additionally, mothers earning between 4,000 SGD to 5,999 SGD had greater methylation than mothers earning more than 6,000 SGD (95% CI [0.013, 0.066], *t*(357) = 2.889, *p* = 0.004) and there was a general decrease in methylation with greater income for the other CpG sites. However, no demographic variables showed a significant contribution to methylation in cg04692870-Probe 1, cg10840135-Probe 4 and cg20046859-Probe 7. Planned contrasts are detailed in Supplementary 1.

### Heritability of methylation at each CpG site

Heritability (R^2^) was calculated to estimate the proportion of variance in CpG methylation values accounted for by genetic variance. Overall mean heritability across probes was higher in the train set (mean *R^2^* = 0.127) than in the test set (mean *R^2^* = 0.101). In the test set, cg15597984-Probe 5 had the strongest heritability (*R^2^* = 0.240), followed by cg04692870-Probe 1 (*R^2^* = 0.168), while cg20046859-Probe 7 had the weakest heritability (*R^2^* = 0.0000475).

### Performance of CpG loci status prediction using multiple linear regression

We conducted multiple linear regression to provide baseline predictions of CpG methylation for comparison with ML approaches. Overall prediction using the linear regression model accounted for an R^2^ of between 0.167 to 0.294 in the train set (mean *R^2^* = 0.233), however in the test set R^2^ values were negative except for cg15597984-Probe 5 (*R^2^* = 0.0747). In terms of RMSE, the test set performed worse as well with a mean RMSE of 0.0346 as compared to the train set (mean RMSE = 0.0318).

### Machine learning models results

#### Elastic net model of genetic features: comparison with heritability

The Elastic net model of genetic features for individual probes (Feature sets 1.2 Individual probes cg04692870-Probe 1, cg09322432-Probe 3, cg10840135-Probe 4, cg15597984-Probe 5, cg20046859-Probe 7) appeared to be equivalent or slightly better in test set performance (*R^2^* = 0.104) than the heritability measure (see Table 3) calculated from the PRS of the significant SNPS (Feature sets 1.2 Individual probes 1, 3–5, 7, *R^2^* = 0.101).

**Table 3:** Heritability estimates (*R^2^*) for CpG methylation values from significant SNPs for each probe and mean heritability for train and test set.

Heritability	cg04692870-Probe 1	cg09322432-Probe 3	cg10840135-Probe 4	cg15597984-Probe 5	cg20046859-Probe 7	Mean (SD)
*R^2^* train set	0.0820	0.172	0.109	0.155	0.121	0.127 (0.0362)
*R^2^* test set	0.168	0.0611	0.0337	0.240	0.0000475	0.101(0.100)

#### Elastic net model of genetic features: comparison with linear regression

Although the Elastic net model using after B-H combined SNPs (Combined Feature set 1.2.9) had comparable performance in the train set in terms of RMSE (mean RMSE = 0.0319, *R^2^* = 0.147) as compared to Linear Regression (mean RMSE = 0.318, *R^2^* = 0.233), it demonstrated superior performance in the test set (RMSE = 0.0298, *R^2^* = 0.093) as compared to the Linear Regression model (RMSE = 0.0346, *R^2^* = −0.250; see Table 4).

**Table 4:** Performance of combined feature set 1.2.9.

Linear Regression	cg04692870-Probe 1	cg09322432-Probe 3	cg10840135-Probe 4	cg15597984-Probe 5	cg20046859-Probe 7	Mean (SD)
RMSE train set	0.065	0.021	0.025	0.032	0.017	0.0318 (0.0193)
*R^2^* train set	0.167	0.294	0.217	0.230	0.258	0.233 (0.0473)
RMSE test set	0.069	0.025	0.029	0.028	0.022	0.0346 (0.0196)
*R^2^* test set	−0.217	−0.403	−0.224	0.0747	−0.480	−0.250 (0.214)

GWAS of 75% samples after B-H correction, each of the 15 significant SNPs across all probe GWASes, mother’s income, household income, accommodation, mother’s highest education level, child’s ethnicity and child’s sex as independent predictors in the prediction of CpG loci methylation beta values for cg04692870-Probe 1, cg09322432-Probe 3, cg10840135-Probe 4, cg15597984-Probe 5, and cg20046859-Probe 7, using linear regression. Performance for each CpG site and average performance across five CpG sites used in prediction were reported.

#### Elastic net model of genetic features: comparison between feature sets

Based on the test set mean R^2^, before B-H GWAS mQTLs for Individual Probes (Feature set 1.1.1, 1.1.3–1.1.5, 1.1.7) had the best performance (*R^2^* = 0.126) among feature sets. Using SNPs from the specific probe GWAS (Feature sets 1.1.1, 1.1.3–1.1.5, 1.1.7 and 1.1.1, 1.1.3–1.1.5, 1.1.7) was superior to models combining SNPs across probe GWASes (Combined Feature sets 1.1.9 and 1.2.9). In terms of RMSE, the GTEx eQTLs (Feature set 2) performed best (RMSE = 0.293), followed by after B-H GWAS mQTLs for Individual Probes (Feature set 1.2.1, 1.2.3–1.2.5, 1.2.7; RMSE = 0.293). RMSE was similar between train and test sets for most feature sets.

#### Comparison between Elastic net and XGBoost algorithms for genetics only feature sets

Based on the test set mean R^2^, XGBoost (Table 5) showed poorer performance as compared to Elastic net (Table 6) for all feature sets. XGBoost showed superior performance in the train set for before B-H Correction mQTLs (Feature sets 1.1) but poorer performance in the test set as compared to the Elastic net models suggesting a greater risk of overfitting in this feature set. Based on the test set mean RMSE, performance appeared to be comparable between XGBoost and Elastic net, with XGBoost demonstrating slightly better results in the before B-H mQTLs (Feature sets 1.1), combined after B-H mQTLs (Combined Feature set 1.2.9), and the 41.5 MB-43.6 MB SNPs (Feature set 3).

**Table 5:** Mean XGBoost model performance on five predicted CpG methylation levels with different sets of genetic features, predicted on an independent testing set.

	XGBoost					
	1. GWAS of 75% of samples				2. GTEx	3. 41.5 Mb-43.6 Mb
	1.1. Before B-H correction		1.2. After B-H correction			
	1.1.1, 1.1.3–1.1.5, 1.1.7. Individual CpG probes	1.1.9. Combined	1.2.1, 1.2.3–1.2.5, 1.2.7. Individual CpG probes	1.2.9. Combined		
Mean RMSE train set (SD)	0.00794 (0.00847)	0.0117 (0.00723)	0.0308 (0.0192)	0.0298 (0.0192)	0.0184 (0.00825)	0.0279 (0.0199)
Mean *R^2^* train set (SD)	0.939 (0.0766)	0.745 (0.261)	0.180 (0.108)	0.246 (0.128)	0.420 (0.322)	0.368 (0.206)
Mean RMSE test set (SD)	0.0308 (0.0201)	0.0302 (0.0178)	0.0295 (0.0164)	0.0296 (0.0174)	0.0302 (0.0194)	0.0291 (0.0176)
Mean *R^2^* test set (SD)	0.0164 (0.138)	0.0211 (0.0689)	0.0470 (0.147)	0.0528 (0.135)	0.0569 (0.0948)	0.100 (0.100)

**Table 6:** Mean Elastic net model performance on five predicted CpG methylation levels with different sets of genetic features, predicted on an independent testing set.

	Elastic net					
	1. GWAS of 75% of samples				2. GTEx	3. 41.5 Mb-43.6 Mb
	1.1. Before B-H correction		1.2. After B-H correction			
	1.1.1, 1.1.3–1.1.5, 1.1.7. Individual CpG probes	1.1.9. Combined	1.2.1, 1.2.3–1.2.5, 1.2.7. Individual CpG probes	1.2.9. Combined		
Mean RMSE train set (SD)	0.0292 (0.0188)	0.0308 (0.0190)	0.0317 (0.0191)	0.0319 (0.0192)	0.0317 (0.0193)	0.0321 (0.0200)
Mean *R^2^* train set (SD)	0.340 (0.0418)	0.192 (0.0629)	0.1474(0.0398)	0.147 (0.0408)	0.148 (0.0528)	0.124 (0.0571)
Mean RMSE test set (SD)	0.0312 (0.0189)	0.0306 (0.0181)	0.0294 (0.0166)	0.0298 (0.0171)	0.0293 (0.0179)	0.0296 (0.0185)
Mean *R^2^* test set (SD)	0.126 (0.101)	0.0640 (0.0442)	0.104 (0.0960)	0.0930 (0.0820)	0.121 (0.0519)	0.119 (0.0502)

#### Comparison between Elastic net models with and without non-genetic and PC features

Based on the test set mean R^2^, the addition of non-genetic and PC factors did not consistently improve performance of Elastic net models. Performance appeared to improve for before B-H Individual Probe mQTLs (Feature set 1.1.1, 1.1.3–1.1.5, 1.1.7, mean R^2^ from 0.126 to 0.141; Table 7) and for after B-H Combined Probe mQTLs (Combined Feature set 1.2.9, mean R^2^ from 0.0930 to 0.0989) while it was worse or similar for other features and when using RMSE for comparison.

**Table 7:** Mean elastic net model performance on five predicted CpG methylation levels with different sets of genetic and non-genetic features, predicted on an independent testing set.

	Elastic net					
	1. GWAS of 75% of samples				2. GTEx	3. 41.5 Mb-43.6 Mb
	1.1. Before B-H correction		1.2. After B-H correction			
	1.1.1, 1.1.3–1.1.5, 1.1.7. Individual CpG probes	1.1.9. Combined	1.2.1, 1.2.3–1.2.5, 1.2.7. Individual CpG probes	1.2.9. Combined		
Mean RMSE train set (SD)	0.0280 (0.0162)	0.0309 (0.0191)	0.0324 (0.0193)	0.0328 (0.0194)	0.0317 (0.0191)	0.0323 (0.0200)
Mean *R^2^* train set (SD)	0.346 (0.0471)	0.193 (0.0683)	0.129 (0.0314)	0.142 (0.0292)	0.150 (0.0568)	0.121 (0.0525)
Mean RMSE test set (SD)	0.0326 (0.0262)	0.0310 (0.0184)	0.0304 (0.0176)	0.0308 (0.0188)	0.0292 (0.0175)	0.0297 (0.0184)
Mean *R^2^* test set (SD)	0.141 (0.0934)	0.0419 (0.0346)	0.0744 (0.0658)	0.0989 (0.0872)	0.121 (0.0493)	0.122 (0.0568)

#### Comparison between XGBoost models with and without non-genetic and PC factors

Overall, the addition of non-genetic and PC factors appeared to be associated with worse or similar performance except for the GTEx eQTLs (Feature set 2, mean R^2^ from 0.0569 to 0.0731; Table 8).

**Table 8:** Mean XGBoost model performance on five predicted CpG methylation levels with different sets of genetic and non-genetic features, predicted on an independent testing set.

	XGBoost					
	1. GWAS of 75% of samples				2. GTEx	3. 41.5 Mb-43.6 Mb
	1.1. Before B-H correction		1.2. After B-H correction			
	1.1.1, 1.1.3–1.1.5, 1.1.7. Individual CpG probes	1.1.9. Combined	1.2.1, 1.2.3–1.2.5, 1.2.7. Individual CpG probes	1.2.9. Combined		
Mean RMSE train set (SD)	0.00656 (0.00308)	0.0172 (0.0124)	0.0301 (0.0182)	0.0294 (0.0176)	0.0283 (0.0163)	0.0279 (0.0193)
Mean *R^2^* train set (SD)	0.936 (0.0604)	0.721 (0.154)	0.214 (0.0606)	0.246 (0.0576)	0.288 (0.0492)	0.348 (0.134)
Mean RMSE test set (SD)	0.0318 (0.0214)	0.0311 (0.0178)	0.0304 (0.0186)	0.0304 (0.0187)	0.0299 (0.0188)	0.0295 (0.0177)
Mean *R^2^* test set (SD)	−0.0168 (0.145)	−0.0507 (0.129)	0.0292 (0.0882)	0.0308 (0.113)	0.0731 (0.0765)	0.0898 (0.0906)

#### Best performing model by CpG probe

The best performing model for CpG cg09322432-Probe 3 and CpG cg15597984-Probe 5 was the Elastic Net model, trained with both genetic and non-genetic features obtained from individual GWAS before B-H correction (Table 9). For CpG cg20046859-Probe 7, the Elastic Net model that was trained with only genetic features obtained from individual GWAS before B-H correction was the best performing model. For CpG cg04692870-Probe 1, the Elastic Net model, trained with genetic features from individual GWAS after B-H correction, achieved the highest R^2^ performance, while the XGBoost model, trained with the same genetic features post B-H correction, demonstrated the best RMSE performance. Lastly, for CpG cg10840135-Probe 4, the Elastic Net model trained with GTEx features sets obtained the best R^2^ performance while the XGBoost model trained with genetic factors from the 41.5 Mb-43.6 Mb range had the best RMSE.

**Table 9:** Comparison of the best performing probes.

	cg04692870-Probe 1, best *R^2^*	cg04692870-Probe 1, best RMSE	cg09322432-Probe 3	cg10840135-Probe 4, best *R^2^*	cg10840135-Probe 4, best RMSE	cg15597984-Probe 5	cg20046859-Probe 7	Mean of best performance/ RMSE (SD)
Model and feature set	Elastic net Ind GWAS after B-H genetics only	XGBoost Ind GWAS after B-H genetics only	Elastic net Ind GWAS uncorrected genetics + environmental	Elastic net GTEx	XGBoost 2 MB genetic only	Elastic net Ind GWAS uncorrected genetics + environmental	Elastic net Ind GWAS uncorrected genetics only	–
No. of Features	2 SNPs (after 10% MAF filtering) + CpG probe	2 SNPs (after 10% MAF filtering) + CpG probe	190 SNPs (after 10% MAF filtering) + 5 PCs + 8 non-genetic factors	548 SNPs (after 10% MAF filtering) + CpG probe	2,406 SNPs (after 10% MAF filtering) + CpG probe	170 SNPs (after 10% MAF filtering) + 5 PCs + 8 non-genetic factors	153 SNPs (after 10% MAF filtering) + CpG probe	–
No. trained	300	300	300	302	305	301	304	–
Hyper parameters	alpha: 0 lambda: 0	objective: reg:squared error eta: 0.003009989 max_depth: 10 colsample_ bytree: 0.5493245 subsample: 0.8383967 nround: 1000	alpha: 0 lambda: 0	alpha: 0 lambda: 1	objective: reg:squared error eta: 0.006558051 max_depth: 1 colsample_ bytree: 0.7361971 subsample: 0.7592239 nround: 924	alpha: 0 lambda: 0	alpha: 0 lambda: 0	–
RMSE train set	0.0646	0.0639	0.0185	0.0249	0.0221	0.0279	0.0152	–
*R^2^* train set	0.104	0.099	0.390	0.109	0.286	0.340	0.327	–
No. tested	101	102	101	102	100	100	102	–
RMSE test set	0.0582	0.0580	0.0176	0.0237	0.0237	0.0256	0.0161	0.0282 (0.017)
*R^2^* test set	0.206	0.179	0.107	0.187	0.155	0.302	0.119	0.184 (0.078)

#### Feature importance of best performing model of each CpG

In general, the features with the top 10 SHAP values (Table 10) were significantly associated with methylation for their respective probes (Supplementary 5), however the most informative features were not necessarily the ones with the highest associations. Five out of the 15 SNPs that were significant after B-H correction could be found among features with the 10 highest SHAP values. For CpG cg04692870-Probe 1, rs133335 GG had the highest SHAP value for both Elastic net and XGBoost models. For the Elastic Net model of CpG cg09322432-Probe 3, rs13447289 had the highest SHAP value. For the Elastic Net model of CpG cg10840135-Probe 4 all top 10 features had the same SHAP value and this included rs133344 CC, which was significantly associated with methylation of CpG cg04692870-Probe 1 but not CpG cg10840135-Probe 4. For the XGBoost model of CpG cg10840135-Probe 4, rs5751045 TT and rs76550409 GG were the top two features and rs76550409 was also significantly associated with CpG cg10840135-Probe 4 after BH correction. For the Elastic Net model of CpG cg15597984-Probe 5, rs1883995 GG had the highest SHAP value and among the top 10 features, rs134906 and rs762995 were both significantly associated with CpG cg15597984-Probe 5 after B-H correction. Lastly, for the Elastic Net model of CpG cg20046859-Probe 7, rs4253623 GG had the highest SHAP value. rs133344 was a top 10 feature for both cg04692870-Probe 1 and cg10840135-Probe 4 and significant after B-H correction in the GWAS study for CpG cg04692870-Probe 1.

**Table 10:** SHAP values of the top features for the best performing model for each *CYP2D6* CpG probe.

CYP2D6 CpG probe	Machine learning algorithm	Features	SHAP value^a^	Features	SHAP value^a^
cg04692870-Probe 1	Elastic net	rs133335 GG	0.0526	rs133335 AG	0.0282
		rs133344 CC	0.0491	rs133344 CA	0.0229
		rs133344 AA	0.0319		
	XGBoost	rs133335 GG	0.00731	rs133344 CC	0.00107
		rs133344 AA	0.00665	rs133344 CA	0.000910
		rs133335 AG	0.00182		
cg09322432-Probe 3	Elastic net	PC2	0.00993	rs133563 GG	0.00255
		PC5	0.00680	rs12628833 TT	0.00255
		PC1	0.00595	rs56103417 CC	0.00216
		rs13447289 TT	0.00470	rs5761074 GG	0.00213
		PC3	0.00295	rs2858226 TT	0.00207
cg10840135-Probe 4	Elastic net	rs5758550 GG	0.000497	rs133331 TT	0.000497
		rs133341 TT	0.000497	rs5751197 TT	0.000497
		rs133344 CC	0.000497	rs129853 TT	0.000497
		rs133333 GG	0.000497	rs133308 GG	0.000497
		rs133332 TT	0.000497	rs133304 TT	0.000497
	XGBoost	rs5751045 TT	0.00130	rs5996145 TC	0.000703
		rs76550409 GG	0.00105	rs5751046 GG	0.000626
		rs9611755 TT	0.000926	rs4822262 TT	0.000597
		rs76392259 GG	0.000806	rs2267432 TT	0.000545
		rs8190368 TT	0.000804	rs2017128 GT	0.000491
cg15597984-Probe 5	Elastic net	rs1883995 GG	0.00676	rs134906 TT	0.00379
		PC4	0.00598	rs80442 CC	0.00374
		PC1	0.00579	rs4820728 TT	0.00363
		rs5995204 TT	0.00453	rs762995 GG	0.00340
		PC5	0.00421	rs7288826 TT	0.00336
cg20046859-Probe 7	Elastic net	rs4253623 GG	0.00498	rs9614421 GG	0.00227
		rs28667050 TT	0.00491	rs5748979 GG	0.00206
		rs2005572 TT	0.00264	rs801581 TT	0.00196
		rs34288001 GG	0.00256	rs4630866 TT	0.00178
		rs117560457 TT	0.00244	rs12159191 GG	0.00174

^a^SHAP values for Elastic Net models are the coefficients of the features of each model, while SHAP values for XGBoost models are calculated for each feature by predicting contributions using a trained XGBoost model.

## Discussion

Here we compare feature selection and machine learning approaches in terms of their relative predictive value for *CYP2D6* CpG loci status. Overall, Elastic net models of genetic features appeared to perform marginally better than heritability estimates and substantially better than Linear Regression and XGBoost models. The addition of non-genetic features appeared to improve performance for some but not all feature sets and probes. The best feature set and ML approach differed substantially between CpG sites, and a number of top variables were identified for each model.

When GWAS was performed to determine SNPs associated with CpG sites, a total of 15 SNPs were identified where several SNPs appeared to influence multiple CpG sites. This suggests that these SNPs are pleiotropic, meaning that they influence multiple traits. After filtering for variants with MAF > 10%, we identified 983 mQTLs from a GWAS of the training set comprising 75% of samples at an uncorrected threshold of *p* < 0.05 and 15 mQTLs at a corrected threshold. We also utilised 548 eQTLs influencing expression of *CYP2D6* across the body from GTEx and 2,406 from a 2 MB range around the gene. That only 15 significant SNPs were left suggesting that there are informative rare variants that are left out by filtering. However, this was necessary in order to prevent errors when running the prediction algorithms but may be a potential limitation of our approach given the importance of rare variants in *CYP2D6*. Even among the significant SNPs where LD pruning had been conducted, there was substantial collinearity, which may explain the relative success of the Elastic net approach especially for cg09322432-Probe 3 and cg15597984-Probe 5, which have a large number of significant mQTLs. Future approaches may infer epigenetics or other expression-based markers in *CYP2D6* based on functional annotation from deep learning algorithms such as Enformer (Avsec et al., 2021) and DeepSEA (Zhou et al., 2018), however RNAseq data on *CYP2D6* did not pass quality checks in our data and it would not have been possible to validate those findings from available data. Additionally, many of our significant SNPs were located beyond the effective range of these algorithms, e.g. rs73885718 more than 1 MB upstream at 22:41386554 (GRCh37). To support this finding, there have been mQTL studies that have shown that the regulation of methylation at a few CpG sites is controlled by SNPs distal to the CpG sites (McVicker et al., 2013; Gaunt et al., 2016).

Various demographic variables contribute to individual CpG sites in varying degrees. Ethnicity influenced methylation at two CpG sites, maternal age had an influence at one site and maternal income had an influence at another site. In particular, Chinese appeared to have greater methylation in cg09322432-Probe 3 and cg15597984-Probe 5, Malays had greater methylation than Indians. This is in keeping with literature on *CYP2D6* allele frequency which is known to vary among ethnic groups (Bradford, 2002) which affects gene expression and function. Chinese have been shown to have a greater frequency of the CYP2D6*10 haplotype (Qian et al., 2013). It may be useful to test for the effects of rare diplotypes on CpG sites in future research. In cg09322432-Probe 3 and cg15597984-Probe 5 the best performing models included non-genetic factors and the PCA components, whereas the best performing models for the other 3 probes were purely SNP-based. This is in keeping with the linear regression results that found associations with ethnicity in these two probes and provides further support for the approach. Age is known to have effects on methylation, and it has been shown epigenetics can be used as a biological clock (McEwen et al., 2020; McGill, 2020; Lahiri et al., 2022). The effect of maternal age on cg17498424-Probe 6, suggests that this could be a heritable epigenetic trait potentially affecting *CYP2D6* function influenced by maternal ‘biological age’. There may be other environmental factors such as smoking during pregnancy that are not captured by ancestry but significantly contribute to variation in methylation (Galanter et al., 2017). Future work could include other environmental factors of GUSTO mothers and children such as diet and exposure to environmental pollutants (Keil and Lein, 2016).

We identified variation in heritability between CpG sites from close to 0 to 0.24 and observed poor performance of a linear regression model. Many epigenetics studies make use of global methylation over a region and our finding of differences between specific CpG sites in the same gene suggests that a finer-grained approach is valuable. It was unexpected that heritability performed better in the test set than the training set with cg04692870-Probe 1 and cg15597984-Probe 5, which will need further replication. That Elastic net was slightly better and XGBoost was substantially worse than heritability estimates for the same features suggests that linear approaches that resolve collinearity may have been more appropriate for this problem.

In the analysis of ML algorithms, Elastic net was superior to XGBoost for identical data and feature sets except for cg04692870-Probe 1 and cg10840135-Probe 4. XGBoost showed superior performance in Kaggle competitions (Chen et al., 2015), and it is unexpected that XGBoost performs poorer than Elastic net in our dataset. A reason XGBoost is not performing as well as expected may be due to the number of samples in training data that is significantly smaller than the number of features which likely causes overfitting. Our dataset also consists of mainly categorical data, which algorithms like LightGBM (Ke et al., 2017) or CatBoost (Dorogush et al., 2018) are better able to support as compared to XGBoost. Elastic Net performing comparably to that of XGBoost may be due to its ability to handle features with collinearity. The grouping effect of Elastic Net groups variables that are highly correlated together, and either drops or retains all of them together (Zou and Hastie, 2005).

Overall, the best performances appeared to be observed from a pure ridge regression for features selected by GWAS at lower or uncorrected thresholds. When additional data such as demographic variables have been found to be associated by conventional statistics, they also improved performance of the model. SNPs from a 1 Mb range around *CYP2D6* CpG sites, which included SNPs that may not be of significance to *CYP2D6* and its methylation, as well as GTEx SNPs appeared to be informative for cg10840135-Probe 4. On one hand inclusion of redundant SNPs could lead to an increase in noise in the CpG methylation prediction model fitting procedure, resulting in less accurate estimation of the coefficients and decreased prediction accuracy. On the other hand, high thresholds may result in the exclusion of informative SNPs that do not survive standard statistical thresholds. Individually, these SNPs would not be considered to be statistically valid mQTLs. However it is an empirical question whether a lower threshold and inclusion of ‘noisier’ or lower confidence features, which probably include several ‘false positives’, improves performance in the test set. Privé et al. (2019) demonstrated that by optimising across a wide range of significance thresholds the generalisability and predictive value of polygenic risk scores (PRS) could be substantially improved. The improved performance of models including lower thresholds has also been demonstrated in deep learning models (Fergus et al., 2018).

We note a number of limitations of the study. This was a relatively small dataset; feature selection was performed within the same set rather than from an external dataset and there was some evidence of overfitting. We mitigated the possibility of data leakage by performing GWAS on only the 75% randomly split training set, such that SNPs significantly associated with the remaining 25% testing set would not be included in the training model. In addition, repeated k-fold cross validation (k = 10, repeats = 5) was used during the model training phase to mitigate the risk of over-fitting. Nonetheless there were differences in performance between the training set and test set that were quite evident when XGBoost was applied in the before B-H GWAS set (Feature set 1.1). This is significant as normal pre-processing methods, such as the method chosen for multiple testing correction for GWAS and its threshold (Loh et al., 2022), could make a difference in models’ predictive performances, and this could be explored in future work. This has implications for the way GWAS studies might be used in the future.

Another limitation was that we were constrained to use MAF < 10% filtering which may result in the loss of informative SNPs that could potentially contribute greatly to ML models. For example, filtering for SNPs for MAF > 10% resulted in the removal of as many as 700 SNPs in the 41.5 Mb – 43.6 Mb range, and these SNPs with rare alleles often have larger phenotypic effects in comparison to common (MAF > 0.05) disease-associated SNPs (Chattopadhyay and Lu, 2020). This could potentially explain the poor performance of some of our ML models. One could also consider training an ensemble model, such as jointly training Elastic Net and XGBoost models in order to prevent overfitting due to the high dimensionality of genetic data (through Elastic Net) while simultaneously exploiting the nonlinear relationships and interaction effects (through XGBoost; Elgart et al., 2022). Lastly, there is a need for a completely independent dataset with more samples that can be used for validation to evaluate our models. Our models should be able to generalise to other datasets to show that they are not overfitted to our data.

This can serve as preliminary work for an improved model for the prediction of methylation using genetic variants in a clinical setting. Due to findings for enrichment of eQTLs at known GWAS risk loci (Nicolae et al., 2010) and overlaps between GWAS risk variants and genomic loci affecting markers of genome regulation (Zhang et al., 2015; Chen et al., 2016; Tehranchi et al., 2016), tools such as PrediXcan and FUSION (Gusev et al., 2016) were implemented for transcriptome-wide association study (TWAS). These tools use the established associations between SNPs and gene expression to impute expression into GWAS samples, which is then used to identify genes relevant to phenotype by testing for their association. This method has been widely used to investigate the role of gene expression in complex traits (Ioannidis et al., 2018; Khawaja et al., 2018; Mancuso et al., 2018; Roselli et al., 2018). These tools represent a powerful approach for interpretation of GWAS findings. Similarly, to these tools, bioinformatics models from this study could allow the development of novel tools to test for association between predicted methylation levels and a phenotype, enabling one to carry out an epigenome-wide association study (EWAS) to identify associations between the trait and imputed epigenome at CpGs across the genome. Existing databases are largely based on expressions and transcriptions due to the amount of research focused on them. It would be powerful if our work can add value to them to accomplish EWAS.

## Data availability statement

Existing datasets are available in a publicly accessible repository. GUSTO datasets were analyzed in this study. This data and R scripts used can be found here: https://figshare.com/projects/Comparing_feature_selection_and_machine_learning_approaches_for_predicting_CYP2D6_methylation_from_genetic_variations/178203.

## Ethics statement

The studies involving humans were approved by Domain Specific Review Board of Singapore National Healthcare Group (D/2009/021) and the Centralised Institutional Review Board of SingHealth (2018/2767/D). The studies were conducted in accordance with the local legislation and institutional requirements. Written informed consent for participation in this study was provided by the participants’ legal guardians/next of kin.

## Author contributions

WJF: analysis and writing was conducted as part of an undergraduate thesis and modified, study design, and writing. HMT: analysis, coding and supervision of machine-learning and writing. MH, BK, ZHC, NYP, and NG: analysis of data using ML under the supervision of HMT, GCYT, and JK. ALT: collection of GUSTO data and writing. HP: extraction of epigenetics data and conceptualisation. DW and VG: guidance on analysis of genomic data. FY, KHT, S-YC, and KYJC: collection of GUSTO data. NR: manuscript preparation, writing and analysis. YHJ: manuscript editing and preparation. RG and ESET: writing and analysis. MM: collection of GUSTO data and support on analysis.
