# Supplementary material for: Comparing feature selection and machine learning approaches for predicting CYP2D6 methylation from genetic variation
Source: Front Neuroinform. 2024 Feb 21;17:1244336. doi: 10.3389/fninf.2023.1244336 (PMC10915285; doi:10.3389/fninf.2023.1244336)
Supplement: Supplementary file 1 [file Table_1.docx]

**Supplementary 1_Additional demographic table and ANCOVA Tables**

**TABLE 1 |** Additional Demographics table

| Sample characteristics | N (%) |
| --- | --- |
| Highest level of education attained |  |
| University | 133 (32.1) |
| National Technical Certificate | 38 (9.20) |
| General Certificate of Education | 104 (25.1) |
| Secondary | 101 (24.4) |
| Primary | 23 (5.56) |
| Not answered | 2 (0.48) |
| Monthly income of household SGD |  |
| <$1,000 | 131 (31.6) |
| $1,000–1,999 | 98 (23.7) |
| $2,000–3,999 | 100 (24.2) |
| $4,000–5,999 | 26 (6.28) |
| ≥$6,000 | 7 (1.69) |
| Not answered | 52 (12.6) |
| Accommodation |  |
| 1-room/ 2-room HDB flat | 17 (4.1) |
| 2-room/ 3-room HDB flat | 81 (19.6) |
| 4-room/ 5-room HDB flat | 234 (56.5) |
| Condominium | 23 (5.56) |
| HDUS/ Executive flat | 11 (2.66) |
| Landed property | 9 (2.17) |
| Others | 2 (0.48) |
| Child sex |  |
| Female | 194 (46.9) |
| Male | 210 (50.7) |

**TABLE 2 |** ANCOVA table for the relationship of demographic variables with cg04692870-Probe 1, adjusted for Mother’s Age at Recruitment

| Source | SS | df | MS | *F* | *p* |
| --- | --- | --- | --- | --- | --- |
| Mother’s Income | 0.01 | 4 | 0.002 | 0.537 | 0.708 |
| Household Income | 0.006 | 4 | 0.002 | 0.343 | 0.849 |
| Accommodation | 0.026 | 6 | 0.004 | 0.957 | 0.455 |
| Mother’s Ethnicity | 0.016 | 2 | 0.008 | 1.769 | 0.172 |
| Mother’s Highest Education | 0.006 | 4 | 0.002 | 0.35 | 0.844 |
| Sex of child | 0.006 | 1 | 0.006 | 1.404 | 0.237 |
| Mother’s Age at Recruitment | 8.201×10^-5^ | 1 | 8.201×10^-5^ | 0.018 | 0.894 |
| Residuals | 1.482 | 321 | 0.005 |  |  |

**TABLE 3 |** ANCOVA table for the relationship of demographic variables with cg07016288-Probe 2, adjusted for Mother’s Age at Recruitment

| Source | SS | df | MS | *F* | *p* |
| --- | --- | --- | --- | --- | --- |
| Mother’s Income | 0.013 | 4 | 0.003 | 2.981 | 0.019 |
| Household Income | 0.003 | 4 | 8.156×10^-4^ | 0.777 | 0.541 |
| Accommodation | 0.004 | 6 | 5.893×10^-4^ | 0.561 | 0.761 |
| Mother’s Ethnicity | 0.004 | 2 | 0.002 | 2.01 | 0.136 |
| Mother’s Highest Education | 0.004 | 4 | 0.001 | 1.066 | 0.373 |
| Sex of child | 8.682×10^-4^ | 1 | 8.682×10^-4^ | 0.827 | 0.364 |
| Mother’s Age at Recruitment | 4.208×10^-5^ | 1 | 4.208×10^-5^ | 0.04 | 0.841 |
| Residuals | 0.339 | 323 | 0.001 |  | 0.019 |

**TABLE 4 |** Simple Contrast table for Mother’s Income with cg07016288-Probe 2, adjusted for Mother’s Age at Recruitment

|  | | | | 95% CI for Mean Difference | | | |  | | | | | | | |
| --- | --- | --- | --- | --- | --- | --- | --- | --- | --- | --- | --- | --- | --- | --- | --- |
| Comparison | | Estimate | | Lower | | Upper | | SE | | df | | *t* | | *p* | |
| <$1,000 - ≥$6,000 | | 0.031 | | 0.006 | | 0.055 | | 0.012 | | 357 | | 2.470 | | 0.014* | |
| $1,000-$1,999 - ≥$6,000 | | 0.028 | | 0.004 | | 0.053 | | 0.013 | | 357 | | 2.260 | | 0.024* | |
| $2,000-$3,999 - ≥$6,000 | | 0.026 | | 0.002 | | 0.051 | | 0.013 | | 357 | | 2.108 | | 0.036* | |
| $4,000-$5,999 - ≥$6,000 | | 0.039 | | 0.013 | | 0.066 | | 0.014 | | 357 | | 2.889 | | 0.004* | |
|  | | | | | | | | | | | | | | | |

**TABLE 5 |** ANCOVA table for the relationship of demographic variables with cg09322432-Probe 3, adjusted for Mother’s Age at Recruitment

| Source | SS | df | MS | *F* | *p* |
| --- | --- | --- | --- | --- | --- |
| Mother’s Income | 3.039×10^-4^ | 4 | 7.596×10^-5^ | 0.144 | 0.965 |
| Household Income | 7.927×10^-4^ | 4 | 1.982×10^-4^ | 0.377 | 0.825 |
| Accommodation | 0.002 | 6 | 3.205×10^-4^ | 0.609 | 0.723 |
| Mother’s Ethnicity | 0.004 | 2 | 0.002 | 3.791 | 0.024 |
| Mother’s Highest Education | 0.002 | 4 | 3.878×10^-4^ | 0.738 | 0.567 |
| Sex of child | 1.453×10^-4^ | 1 | 1.453×10^-4^ | 0.276 | 0.599 |
| Mother’s Age at Recruitment | 6.148×10^-4^ | 1 | 6.148×10^-4^ | 1.169 | 0.28 |
| Residuals | 0.17 | 323 | 5.258×10^-4^ |  |  |

| **TABLE 6 \|** Simple Contrast table for mother’s ethnicity with cg09322432-Probe 3, adjusted for Mother’s Age at Recruitment | | | | | | | | | | | | | | | |
| --- | --- | --- | --- | --- | --- | --- | --- | --- | --- | --- | --- | --- | --- | --- | --- |
|  | | | | 95% CI for Mean Difference | | | |  | | | | | | | |
| Comparison | | Estimate | | Lower | | Upper | | SE | | df | | *t* | | *p* | |
| Chinese - Malay | | 0.007 | | 0.002 | | 0.012 | | 0.003 | | 373 | | 2.658 | | 0.008* | |
| Chinese - Indian | | 0.007 | | 6.355×10^-4^ | | 0.014 | | 0.003 | | 373 | | 2.155 | | 0.032 | |
| Malay - Indian | | -0.002 | | -0.007 | | 0.008 | | 0.004 | | 373 | | 0.075 | | 0.940 | |
|  | | | | | | | | | | | | | | | |

**TABLE 7 |** ANCOVA table for the relationship of demographic variables with cg10840135-Probe 4, adjusted for Mother’s Age at Recruitment

| Source | SS | df | MS | *F* | *p* |
| --- | --- | --- | --- | --- | --- |
| Mother’s Income | 0.002 | 4 | 4.337×10^-4^ | 0.595 | 0.667 |
| Household Income | 7.267×10^-4^ | 4 | 1.817×10^-4^ | 0.249 | 0.91 |
| Accommodation | 0.002 | 6 | 3.020×10^-4^ | 0.414 | 0.87 |
| Mother’s Ethnicity | 0.003 | 2 | 0.001 | 1.717 | 0.181 |
| Mother’s Highest Education | 9.340×10^-4^ | 4 | 2.335×10^-4^ | 0.32 | 0.864 |
| Sex of child | 0.002 | 1 | 0.002 | 2.085 | 0.15 |
| Mother’s Age at Recruitment | 5.265×10^-4^ | 1 | 5.265×10^-4^ | 0.722 | 0.396 |
| Residuals | 0.236 | 323 | 7.295×10^-4^ |  |  |

**TABLE 8 |** ANCOVA table for the relationship of demographic variables with cg15597984-Probe 5, adjusted for Mother’s Age at Recruitment

| Source | SS | df | MS | *F* | *p* |
| --- | --- | --- | --- | --- | --- |
| Mother’s Income | 0.002 | 4 | 4.589×10^-4^ | 0.434 | 0.784 |
| Household Income | 0.004 | 4 | 9.209×10^-4^ | 0.87 | 0.482 |
| Accommodation | 0.007 | 6 | 0.001 | 1.06 | 0.387 |
| Mother’s Ethnicity | 0.012 | 2 | 0.006 | 5.48 | 0.005 |
| Mother’s Highest Education | 0.002 | 4 | 4.792×10^-4^ | 0.453 | 0.77 |
| Sex of child | 0.003 | 1 | 0.003 | 3.134 | 0.078 |
| Mother’s Age at Recruitment | 0.001 | 1 | 0.001 | 1.324 | 0.251 |
| Residuals | 0.342 | 323 | 0.001 |  |  |

| **TABLE 9 \|** Simple Contrast table for mother’s ethnicity with cg15597984-Probe 5, adjusted for Mother’s Age at Recruitment | | | | | | | | | | | | | | | |
| --- | --- | --- | --- | --- | --- | --- | --- | --- | --- | --- | --- | --- | --- | --- | --- |
|  | | | | 95% CI for Mean Difference | | | |  | | | | | | | |
| Comparison | | Estimate | | Lower | | Upper | | SE | | df | | *t* | | *p* | |
| Chinese - Malay | | 0.006 | | -0.002 | | 0.014 | | 0.004 | | 373 | | 1.567 | | 0.118 | |
| Chinese - Indian | | 0.018 | | 0.008 | | 0.027 | | 0.005 | | 373 | | 3.540 | | < .001* | |
| Malay - Indian | | 0.011 | | 7.822 x 10^-4^ | | 0.022 | | 0.005 | | 373 | | 2.110 | | 0.036* | |
|  | | | | | | | | | | | | | | | |

**TABLE 10 |** ANCOVA table for the relationship of demographic variables with cg17498424-Probe 6, adjusted for Mother’s Age at Recruitment

| Source | SS | df | MS | *F* | *p* |
| --- | --- | --- | --- | --- | --- |
| Mother’s Income | 5.904×10^-4^ | 4 | 1.476×10^-4^ | 0.334 | 0.855 |
| Household Income | 0.001 | 4 | 2.526×10^-4^ | 0.572 | 0.683 |
| Accommodation | 0.001 | 6 | 2.436×10^-4^ | 0.552 | 0.769 |
| Mother’s Ethnicity | 0.002 | 2 | 0.001 | 2.748 | 0.066 |
| Mother’s Highest Education | 1.108×10^-4^ | 4 | 2.769×10^-5^ | 0.063 | 0.993 |
| Sex of child | 5.039×10^-5^ | 1 | 5.039×10^-5^ | 0.114 | 0.736 |
| Mother’s Age at Recruitment | 0.003 | 1 | 0.003 | 6.707 | 0.01 |
| Residuals | 0.141 | 320 | 4.417×10^-4^ |  |  |

**TABLE 11 |** ANCOVA table for the relationship of demographic variables with cg20046859-Probe 7, adjusted for Mother’s Age at Recruitment

| Source | SS | df | MS | *F* | *p* |
| --- | --- | --- | --- | --- | --- |
| Mother’s Income | 7.032×10^-4^ | 4 | 1.758×10^-4^ | 0.527 | 0.716 |
| Household Income | 8.472×10^-4^ | 4 | 2.118×10^-4^ | 0.635 | 0.638 |
| Accommodation | 0.002 | 6 | 2.809×10^-4^ | 0.841 | 0.539 |
| Mother’s Ethnicity | 1.763×10^-4^ | 2 | 8.815×10^-5^ | 0.264 | 0.768 |
| Mother’s Highest Education | 0.001 | 4 | 2.736×10^-4^ | 0.82 | 0.513 |
| Sex of child | 9.664×10^-5^ | 1 | 9.664×10^-5^ | 0.29 | 0.591 |
| Mother’s Age at Recruitment | 2.571×10^-5^ | 1 | 2.571×10^-5^ | 0.077 | 0.782 |
| Residuals | 0.108 | 323 | 3.338×10^-4^ |  |  |
