# Supplementary material for: Comparing feature selection and machine learning approaches for predicting CYP2D6 methylation from genetic variation
Source: Front Neuroinform. 2024 Feb 21;17:1244336. doi: 10.3389/fninf.2023.1244336 (PMC10915285; doi:10.3389/fninf.2023.1244336)
Supplement: Supplementary file 4 [file Table_4.docx]

**Supplementary 5: Top SNPs Regression**

Table A: Mean methylation values of the top SNPs identified for each *CYP2D6* CpG probes, and ANOVA results of the top SNPs against their respective *CYP2D6* CpG probes methylation values.

| CYP2D6 CpG Probe | Machine Learning Algorithm | SNP ID (Major allele homozygote, Heterozygote, Minor allele homozygote) | Major allele homozygote  Mean methylation (SD) | Heterozygote  Mean methylation (SD) | Minor allele homozygote  Mean methylation (SD) | ANOVA, F (p-value) |
| --- | --- | --- | --- | --- | --- | --- |
| cg04692870-Probe 1 | Elastic Net and XGBoost | rs133335 (GG, AG, AA) | 0.600 (0.0686) | 0.570 (0.0545) | 0.502 (0.0576) | 15.1 (5.69x10^-7^) |
|  |  | rs133344 (AA, CA, CC) | 0.593 (0.0674) | 0.565 (0.0549) | 0.465 (0.0622) | 7.90 (4.36x10^-5^) |
| cg09322432-Probe 3 | Elastic Net | rs13447289 (CC, TC, TT) | 0.900 (0.0214) | 0.897 (0.0246) | 0.867 (0.0375) | 9.18 (0.000100) |
|  |  | rs133563 (AA, GA, GG) | 0.896 (0.0246) | 0.902 (0.0195) | 0.904 (0.0277) | 2.71 (0.0684) |
|  |  | rs5761074 (AA, GA, GG) | 0.901 (0.0202) | 0.893 (0.0257) | 0.880 (0.0382) | 8.53 (0.000200) |
|  |  | rs12628833 (CC, TC, TT) | 0.899 (0.0228) | 0.892 (0.0246) | 0.868 (0.0306) | 3.52 (0.0310) |
|  |  | rs56103417 (AA, CA, CC) | 0.900 (0.0213) | 0.896 (0.0244) | 0.885 (0.0375) | 3.40 (0.0348) |
|  |  | rs285822 (CC, TC, TT) | 0.901 (0.0226) | 0.895 (0.0233) | 0.878 (0.0223) | 7.74 (0.000500) |
| cg10840135-Probe 4 | Elastic Net | rs5758550 (AA, GA, GG) | 0.722 (0.0560) | 0.731 (0.0262) | 0.758 (0.0147) | 4.92 (0.00790) |
|  |  | rs133341 (CC, TC, TT) | 0.723 (0.0260) | 0.730 (0.0261) | 0.758 (0.0147) | 4.51 (0.0117) |
|  |  | rs133344 (AA, CA, CC) | 0.723 (0.0260) | 0.730 (0.0261) | 0.758 (0.0147) | 4.51 (0.0117) |
|  |  | rs133333 (AA, GA, GG) | 0.722 (0.0260) | 0.731 (0.0262) | 0.758 (0.0147) | 4.92 (0.00790) |
|  |  | rs133332 (CC, TC, TT) | 0.722 (0.0260) | 0.731 (0.0262) | 0.758 (0.0147) | 4.92 (0.00790) |
|  |  | rs133331 (CC, TC, TT) | 0.723 (0.0260) | 0.730 (0.0261) | 0.758 (0.0147) | 4.51 (0.0117) |
|  |  | rs5751197 (CC, TC, TT) | 0.723 (0.0260) | 0.730 (0.0261) | 0.758 (0.0147) | 4.51 (0.0117) |
|  |  | rs129853 (CC, TC, TT) | 0.723 (0.0260) | 0.730 (0.0261) | 0.758 (0.0147) | 4.51 (0.0117) |
|  |  | rs133308 (AA, GA, GG) | 0.722 (0.0260) | 0.731 (0.0262) | 0.758 (0.0147) | 4.92 (0.00790) |
|  |  | rs133304 (CC, TC, TT) | 0.723 (0.0260) | 0.730 (0.0261) | 0.758 (0.0147) | 4.51 (0.0117) |
|  | XGBoost | rs5751045 (TT, CT, CC) | 0.730 (0.0243) | 0.715 (0.0271) | 0.707 (0.0213) | 15.3 (4.69x10^-7^) |
|  |  | rs76550409 (GG, AG, AA) | 0.730 (0.0252) | 0.716 (0.0249) | 0.706 (0.0244) | 16.1 (2.35x10^-7^) |
|  |  | rs9611755 (TT, CT, CC) | 0.733 (0.0257) | 0.718 (0.0255) | 0.714 (0.0213) | 15.3 (4.49x10^-7^) |
|  |  | rs76392259 (GG, AG, AA) | 0.736 (0.0262) | 0.723  (0.0236) | 0.711 (0.0243) | 21.6 (1.74x10^-9^) |
|  |  | rs8190368 (TT, TG, GG) | 0.717 (0.0300) | 0.725 (0.0245) | 0.733 (0.0241) | 6.62 (0.00150) |
|  |  | rs5996145 (CC, TC, TT) | 0.720 (0.0269) | 0.728 (0.0247) | 0.720 (0.0284) | 3.97 (0.0198) |
|  |  | rs5751046 (AA, GA, GG) | 0.720 (0.0256) | 0.723 (0.0258) | 0.732 (0.0263) | 5.64 (0.00390) |
|  |  | rs4822262 (CC, TC, TT) | 0.726 (0.0245) | 0.725 (0.0282) | 0.715 (0.0266) | 2.58 (0.0772) |
|  |  | rs2267432 (TT, CT, CC) | 0.720 (0.0251) | 0.733 (0.0259) | 0.743 (0.0300) | 10.0 (6.12x10^-5^) |
|  |  | rs2017128 (TT, GT, GG) | 0.715 (0.0253) | 0.730 (0.0262) | 0.728 (0.0226) | 12.2 (7.81x10^-6^) |
| cg15597984-Probe 5 | Elastic Net | rs1883995 (AA, GA, GG) | 0.741 (0.0319) | 0.732 (0.0390) | 0.705 (0.0295) | 4.04 (0.0185) |
|  |  | rs5995204 (CC, TC, TT) | 0.742 (0.0324) | 0.733 (0.0372) | 0.716 (0.0223) | 4.19 (0.0160) |
|  |  | rs134906 (CC, TC, TT) | 0.744 (0.0335) | 0.739 (0.0306) | 0.715 (0.0345) | 9.89 (6.96x10^-5^) |
|  |  | rs80442  (AA, CA, CC) | 0.738 (0.0339) | 0.747 (0.0314) | 0.720 (0.0168) | 2.20 (0.113) |
|  |  | rs762995 (AA, GA, GG) | 0.754 (0.0252) | 0.735 (0.0339) | 0.713 (0.0315) | 32.2 (2.22x10^-13^) |
|  |  | rs7288826 (GG, TG, TT) | 0.740 (0.0330) | 0.738 (0.0353) | 0.719 (0.0346) | 1.54 (0.217) |
|  |  | rs4820728 (CC, TC, TT) | 0.743 (0.0312) | 0.733 (0.0350) | 0.720 (0.0558) | 4.33 (0.0140) |
| cg20046859-Probe 7 | Elastic Net | rs28667050 (CC, TC, TT) | 0.879 (0.0176) | 0.880 (0.0202) | 0.847 (0.0202) | 3.31 (0.0378) |
|  |  | rs4253623 (AA, GA, GG) | 0.878 (0.0177) | 0.886 (0.0196) | 0.876 (0.00387) | 4.43 (0.0127) |
|  |  | rs4630866 (CC, TC, TT) | 0.880 (0.0182) | 0.877 (0.0183) | 0.872 (0.0163) | 1.27 (0.281) |
|  |  | rs34288001 (AA, GA, GG) | 0.878 (0.0188) | 0.882 (0.0159) | 0.885 (0.0137) | 1.45 (0.235) |
|  |  | rs2005572 (CC, TC, TT) | 0.880 (0.0173) | 0.877 (0.0209) | 0.869 (0.0155) | 2.09 (0.125) |
|  |  | rs117560457 (CC, TC, TT) | 0.880 (0.0181) | 0.876 (0.0179) | 0.866 (0.0219) | 2.76 (0.0648) |
|  |  | rs5748979 (AA, GA, GG) | 0.883 (0.0174) | 0.876 (0.0180) | 0.870 (0.0189) | 8.80 (0.000200) |
|  |  | rs801581 (CC, TC, TT) | 0.882 (0.0181) | 0.880 (0.0172) | 0.867 (0.0195) | 8.86 (0.000200) |
|  |  | rs9614421 (AA, GA, GG) | 0.879 (0.0181) | 0.878 (0.0185) | 0.872 (0.0216) | 0.598 (0.551) |
|  |  | rs12159191 (AA, GA, GG) | 0.880 (0.0181) | 0.877 (0.0179) | 0.873 (0.0217) | 1.41 (0.247) |
