# Supplementary material for: Comparing feature selection and machine learning approaches for predicting CYP2D6 methylation from genetic variation
Source: Front Neuroinform. 2024 Feb 21;17:1244336. doi: 10.3389/fninf.2023.1244336 (PMC10915285; doi:10.3389/fninf.2023.1244336)
Supplement: Supplementary file 5 [file Data_Sheet_1.docx]

**Supplementary 2_ Links to repositories**

1. Link to Figshare repository:

<https://figshare.com/projects/Comparing_feature_selection_and_machine_learning_approaches_for_predicting_CYP2D6_methylation_from_genetic_variations/178203>

1. Scripts for genotype imputation

^1^ <https://bitbucket.org/sics-sb/gusto-imputation.git>

^2^ <https://github.com/natacha-beck/imputePrepSanger>
